# Search for Information-Bearing Components in Neural Data

**DOI:** 10.1371/journal.pone.0099793

**Published:** 2014-06-16

**Authors:** Meng Hu, Hualou Liang

**Affiliations:** School of Biomedical Engineering, Science & Health Systems, Drexel University, Philadelphia, Pennsylvania, United State of America; Universiteit Gent, Belgium

## Abstract

Multivariate empirical mode decomposition (MEMD) is an important extension of EMD, suitable for processing multichannel data. It can adaptively decompose multivariate data into a set of intrinsic mode functions (IMFs) that are matched both in number and in frequency scale. This method is thus holds great potential for the analysis of multi- channel neural recordings as it is capable of ensuring all the intrinsic oscillatory modes are aligned not only across channels, but also across trials. Given a plethora of IMFs derived by MEMD, a question of significant interest is how to identify which IMFs contain information, and which IMFs are noise. Existing methods that exploit the dyadic filter bank structure of white noise decomposition are insufficient since the IMFs do not always adhere to the presumed dyadic relationship. Here we propose a statistical procedure to identify information-bearing IMFs, which is built upon MEMD that allows adding noise as separate channels to serve as a reference to facilitate IMF identification. In this procedure, Wasserstein distance is used to measure the similarity between the reference IMF and that from data. Simulations are performed to validate the method. Local field potentials from cortex of monkeys while performing visual tasks are used for demonstration.

## Introduction

Neural data are inevitably contaminated by noise. The presence of noise can adversely impact the statistical analysis of data, thus impede our ability to extract meaningful information from noisy data. While much effort has been devoted directly to the removal of noise, e.g. denoising [1], adding noise to data has been increasingly used to help data analysis (e.g., sensitivity analysis of noise robustness [2]), and enhance the perception of otherwise undetectable stimuli via the mechanism of stochastic resonance [3]. In this contribution, we introduce a novel statistical procedure to determine the information-bearing components in neural data. Our method is developed within the general framework of multivariate empirical mode decomposition (MEMD) [4] that allows for adding noise as separate channels, where the effect of the added noise is to provide a reference to facilitate signal identification.

Multivariate empirical mode decomposition (MEMD) is an important extension of empirical mode decomposition (EMD), suitable for analyzing multichannel data. Empirical mode decomposition (EMD) [5] is a fully adaptive, data-driven method that decomposes a time series into a finite set of amplitude-frequency modulated components, referred to as intrinsic mode functions (IMFs). The last decade has witnessed remarkable success of EMD in a variety of applications; it is however limited to univariate (single-channel) data analysis. The availability of simultaneous multi-channel data presents important analysis challenges and calls for multivariate extension of EMD. Among several extensions available [6]-[10], MEMD is thus far a rather generic multivariate extension, and has the ability to find common oscillatory modes/scales across different IMFs within multivariate data. Given a plethora of IMFs derived by MEMD, it is natural to ask: how to identify which IMFs contain information and which IMFs are noise? This is still an unsolved problem that we will directly address with the use of noise.

The use of noise in data analysis has long been known. There are only a few such studies that are relevant to EMD analysis. Broadly, there are two approaches to utilize noise for EMD analysis. One is to assign statistical significance of information content to IMF components from any noisy data by exploiting numerical observations that (1) EMD of white noise acts essentially as a dyadic filter [11], and (2) all the IMFs obtained from white noise follow a normal distribution [12]. The second approach is to improve the EMD method by directly adding noise to the data. Previous attempt has been made to add noise of infinitesimal amplitude to the data in order to make the EMD operable [13]. Wu and Huang [14] explored the benefit of dyadic filter bank structure of EMD for white noise, and proposed ensemble EMD (EEMD) in which multiple realizations of white noise are added to the data before EMD is applied. The effect of the added white noise is to provide a uniformly distributed reference scale, which enables EMD to preserve the dyadic property and hence reduce the chance of mode mixing. The mode-mixing occurs when a single IMF contains multiple oscillatory modes and/or a single mode resides in multiple IMFs, which in many cases may obscure the physical meaning of IMFs. Given the random effect of noise in multiple realizations, added noise is eventually canceled out in the ensemble average. Similar to EEMD, recently proposed noise-assisted MEMD (NA_MEMD) [15] also makes use of the dyadic property to reduce the mode-mixing problem; yet it adds white noise as extra channels and hence only a single sweep of MEMD is applied.

In this contribution, we introduce a novel statistical procedure to determine the information-bearing components from neural data. Our method is developed within the framework of MEMD that allows adding noise as separate channels, which in turn serve as a reference to facilitate IMF component identification in the decomposition. In this method, Wasserstein distance [16] is used to measure the similarity between the reference IMF from noise and the IMF from data. It is efficiently estimated based on the rank-order statistics, thus is robust to outliers. Confidence intervals for Wasserstein distance is assessed by the Monte-Carlo method. Our proposed method is demonstrated by both computer simulations and real cortical field potentials data collected from visual cortex of a macaque monkey while performing a generalized flash suppressing (GFS) task [17].

This article is organized as follows. In Section 2, we describe the basic MEMD, and the proposed signal identification approach. In Section 3, we present simulation examples and the analysis of neural data to demonstrate the application of our approach. Section 4 concludes the paper with discussion of some practical issues relating to applications.

## Materials and Methods

### Experiment and neurophysiological recording

Generalized flash suppressing (GFS) is a visual illusion, in which a salient visual stimulus could be rendered invisible despite continuous retinal input, providing a rare opportunity to study neural mechanisms directly related to perception [17]. In the GFS task, as soon as a monkey gained fixation for about 300 msec, the target stimulus indicated by a red disk was presented. At 1400 msec after the target onset, small random-moving dots appeared as the surroundings. With the immediate presentation of the surroundings, the red disk could be rendered subjectively invisible. If the target stimulus disappeared from perception, the monkey was trained to release a lever; otherwise, monkey was to hold the lever. Therefore, based on the responses of the animal, the trial was classified as either ‘‘visible’’ or ‘‘invisible’’. Note that the stimuli in these two conditions were physically identical.

Multi-electrode local field potential (LFP) recordings were simultaneously collected from multiple cortical areas V1, V2, and V4 while monkeys performed the GFS task. The data were obtained by band-pass filtering the full bandwidth signal between 1 and 500 Hz, and then resampled at 1000 Hz. In this report, we use the LFP neural recordings from area V1 to illustrate the effectiveness of our method.

### Multivariate empirical mode decomposition (MEMD)

MEMD is the multivariate extension of EMD. The EMD [5] acts as a fully adaptive data-driven method, which decomposes a time series into a finite set of scale-dependent IMFs, representing its inherent oscillatory modes. Specifically, for a time series 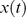, all the local extrema are first identified, and then two envelopes 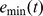 and 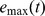 are obtained by interpolating between local maxima (resp. minima) to compute the local mean 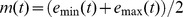. The procedure iterates on the detail 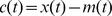 until it becomes an IMF (the symmetric envelopes and the same numbers of zero-crossing and local extrema, differing at most by one). The residue obtained by removing IMFs from raw signal is subject to the above procedure for the next IMF until the monotonic residue is left. Hence, A time series 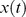 can be expressed as: 
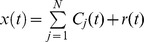
, where 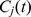, *j*  =  1,…, *N* are the IMFs, and *r*(*t*) is the residue.

In neuroscience, as in many other fields of science and engineering, data of interest are often collected in the form of multiple simultaneous time series. Therefore, extension of EMD to multivariate time series is required for accurate data-driven time-frequency representation. Recently MEMD has been proposed to extend the application of EMD to multivariate time series, with the ability to find common oscillatory modes/scales across different IMFs within multivariate time series [4]. An important step in the MEMD method is the calculation of local mean, as the concept of local extrema is not well defined for multivariate signals. To deal with this problem, MEMD projects the multivariate signal along different directions to generate the multiple multidimensional envelopes; these envelopes are then averaged to obtain the local mean. For an *n*-variable signal, the MEMD algorithm is briefly summarized as follows:

(i) Construct the suitable point set for sampling on an (*n*-1)-sphere;

(ii) Compute a projection 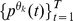 of the multivariate input data 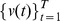 along a direction vector 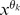 for all *k* giving 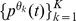;

(iii) Locate the time points 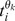 corresponding to maxima of the set of projected signal 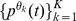;

(iv) Interpolate [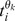,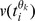] to acquire multivariate envelope curves 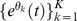;

(v) Calculate the mean *m*(*t*) of the envelope curves for a set of *K* direction vectors, 
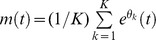
;

(vi) Iterate on the detail 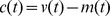 until it becomes an IMF. The above procedure is applied to the residue 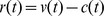.

The stoppage criterion for multivariate IMF is similar to that for univariate IMFs except that the equality constraint for number of extrema and zero crossings is not imposed, as the extrema cannot be properly defined for multivariate signal.

The noise-assisted MEMD (NA_MEMD) [15] is essentially the same as the MEMD except the innovative use of noise in such a way that white noise is added as *separate* channel(s). The NA_ MEMD is therefore distinctly different from the ensemble empirical mode decomposition (EEMD) [14] in which multiple realizations of white noise are added to the data before EMD is applied. As such, the NA_MEMD, while retaining all the desired properties of MEMD, can effectively eliminate the interference of noise in EEMD and reduce the mode mixing problem in the existing EMD algorithms. The efficacy of the NA_MEMD method has been recently demonstrated via both simulations and applications of EEG data [21].

Our proposed method is built upon the NA_MEMD approach that allows adding noise as separate channels, which can be used as a reference to facilitate IMF component identification in the decomposition. In this method, Wasserstein distance is used to measure the similarity between the reference IMF from noise and the IMF from data. In the following, we briefly describe the Wasserstein distance with emphasis on its efficient estimation based on the rank-order statistics.

### Wasserstein distance

Wasserstein distance is a general distance measure between any probability distributions. As a metric, it respects fundamental metric properties such as non-negativity, symmetry, the triangle inequality and identity of indiscernibles, thus has a plenitude of applications in various fields [18]-[19]. The Wasserstein distance between two probability measures or distribution functions 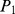 and 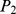 on a sample space Ω is defined as:
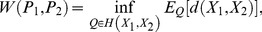
(1)where 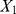 and 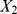 are random variables with probability distributions 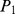 and 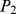 respectively, 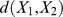 is the distance between 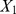 and 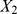, 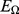 is the expectation operation over the sample space Ω, and the infimum is over all joint distributions 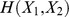 of 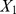
and 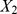.

The Wasserstein distance is commonly given by the solution of an optimal transportation problem [20]. It measures the amount of work, or distance times probability mass transported, that is needed to transform 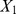 into 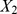, weighted according to an exponent *k*. Formally, it is given by the functional [18]:
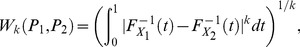
(2)where 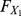
and 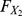 are the cumulative distribution functions of 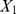 and 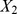 respectively, and 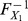 and 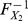 represent their respective inverse functions. In practice, the 1^st^ Wasserstein distance (*k* = 1, also dubbed as earth mover’s distance [20]) is perhaps the most commonly used measure of distance between two probability distributions, especially in computer science.

To estimate the cumulative distribution function (CDF), we follow an intuitively empirical method based on the rank-order statistics, which is given as follows:
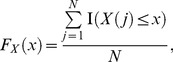
(3)


where I refers to the indicator function and *N* is the length of time series *X*. The inverse CDF can then be calculated as:
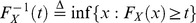
(4)


We note that the estimation of the CDF and its inverse are conducted based on the rank-order statistics, our approach has the advantage of being robust to the outliers in the data.

### Statistical identification of informative components

In this study we propose a statistical procedure to identify significant information-bearing IMFs. Our procedure is built upon the NA_MEMD framework in which white noise is added as separate channel(s) together with the original data to form a composite multivariate time series, on which MEMD is performed. The idea here is to use the added noise channel(s) to serve as a reference for identification of the informative IMFs in the decomposition. The proposed approach mainly consists of two steps: (1) data preprocessing, and (2) adding noise to the data and statistical identification of informative IMF components.

#### Data preprocessing

Neural data possess some unique characteristics that must be considered when MEMD is performed: (1) neural data are often collected over certain time period from multiple channels across many trials, which can be represented as a three-dimensional matrix, i.e., Time × Channels × Trials, on which the MEMD cannot be directly applied, and (2) neural recordings are usually of high degree of variability, typically collected over many trials spanning from days to months, or even years, which has deleterious impact upon the final decomposition of MEMD when projecting the data in multidimensional space. Therefore, two important preprocessing steps [22] should be taken before applying the MEMD to neural data. First, high-dimensional neural data (e.g., Time × Channels × Trials) is reshaped into such a two-dimensional time series as Time × [Channels × Trials] before submitted to the MEMD analysis. During the reshaping process, it is required to have the same rows (Time) in the resulting matrix. Note that it is an important step to make sure that all the IMFs be aligned not only across channels, but also across trials. Second, in order to reduce the variability among neural recordings, each individual time series is normalized against its temporal standard deviation before the MEMD is applied, and subsequently restore the standard deviations to the corresponding IMFs after the MEMD.

#### Statistical identification procedure

With the preprocessed data, white noise is added as the extra channel(s) to form a composite data for decomposition of MEMD. To differentiate the information-bearing IMFs from noisy IMFs in the data, the IMFs from the separate noise channels are used as a reference. The idea is to determine the extent to which the IMFs obtained from the data differ from those obtained from the reference noise channels. To implement this idea, we use Wasserstein distance to assess the similarity between the reference IMF from noise channels and the IMF from data.

To establish a confidence interval for the Wasserstein distance, we employ a Monte-Carlo technique. To do so, we build a null distribution of the Wasserstein distance between the IMFs derived from reference noise. The generation of such null distribution greatly benefits from the observation that there is no limit on the number of noise channels used in the NA_MEMD [21]. From the resulting distribution at individual scales, we determine the 0.025 and 0.975 quantiles. In this way, we use the null distribution to define the 95% confidence interval for the Wasserstein distance. An IMF obtained from the data is considered to be significantly (*p*<0.05) informative if its Wasserstein distance to the noise reference IMFs is outside the 95% confidence interval. Our approach is summarized as follows:

1. Preprocess the *n*-channel (*n* ≥ 1) time series data as described in the text.
2. Generate *m*-channel (*m* ≥ 1) uncorrelated Gaussian white noise time series (noise reference channels) of the same length as that of the data, together with the *n*-channel preprocessed data to construct composite (*n+m*)-channel data.
3. Perform MEMD on the composite (*n+m*)-channel data.
4. Construct a null distribution of the Wasserstein distance between the IMFs from m-channel noise reference to establish the 95% confidence interval at each scale.
5. At a given scale, calculate the Wasserstein distance between IMFs obtained from the *n*-channel data and those from the m-channel noise reference.
6. An IMF obtained from the data is considered to be significantly informative (*p* < 0.05) if the Wasserstein distance obtained in (5) is outside the 95% confidence interval established in (4).

In practice, the Wasserstein distance is computed with a short-time sliding window to account for the data nonstationarity, and to detect ephemeral signals contained in the data. Additionally, there is an important question in applying the above procedure for the data analysis: how much noise in the reference channels should be used relative to the input data? Whereas the method has no limit on the number of noise channels used, excessive noise levels can compromise the data-driven ability of the method [21]. As such, we choose in this study the noise variance of 6% relative to the input data, which is within 2%-10% of the variance of data as suggested [21].

## Results

In this section, we first perform simulations to verify the effectiveness of our approach in identifying the informative components from the data, and then apply it to real local field potential data to demonstrate the application of the method. In the following simulations, unless otherwise specified, we generate 15-channel uncorrelated Gaussian white noise time series of the same length as that of the data as the noise reference channels. The noise variance of 6% relative to the input data is used.

### Simulations

#### Identification of information-bearing components in the simulated data

In this simulation, a synthetic trivariate time series X, Y and Z is generated with three sinusoidal waves of 12 Hz, 26 Hz and 50 Hz, respectively. One sinusoidal wave of 50 Hz is designed present on all three channels, whereas the remaining two sinusoidal waves, one of 12 Hz appears in both channel X and Y, and another of 26 Hz appears in both channel X and Z. Randomly generated white noise is added to each channel.

**Figure 1:**
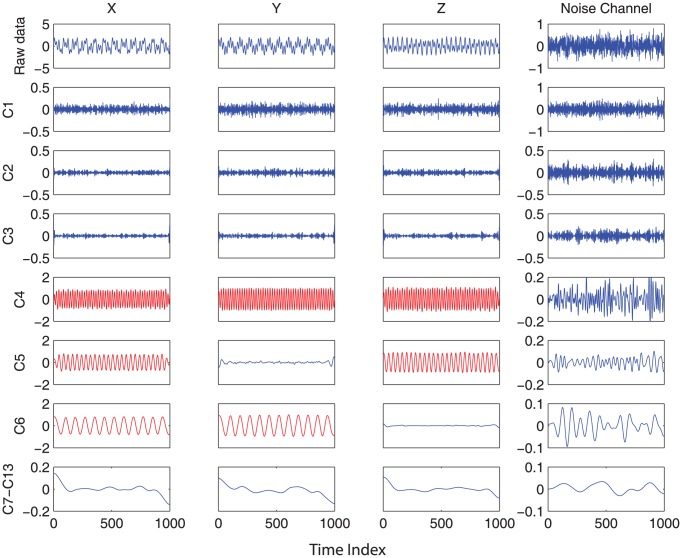
MEMD decomposition of composite time series consisting of the original 3-channel synthetic data [X Y Z] and noise reference channels. For the purpose of clarity, only one noise channel is shown. All the sine waves with distinct frequencies (red) are correctly obtained via MEMD. At the same time, the IMFs obtained from the added noise channel provide the reference for statistical identification of significant IMFs.

First, the white noises are generated as the additional, separate channels to the preprocessed trivariate time series to construct the composite data. Such composite data (shown in the top row of Fig. 1 where only one channel of noise is included for illustration), when subjected to the MEMD, yields six IMF components (labeled as C1-C6 on the left of Fig.1) and residuals which are summed over the remaining IMFs for the purpose of clarity. We can see that each IMF carries a single frequency oscillatory mode, confirming the excellent alignment of common scales across different components derived from the MEMD. In addition, all the sine waves with distinct frequencies in the simulation are correctly shown in the decomposition (highlighted in red). Still, other components remain unknown as to whether they contain significant information.

**Figure 2:**
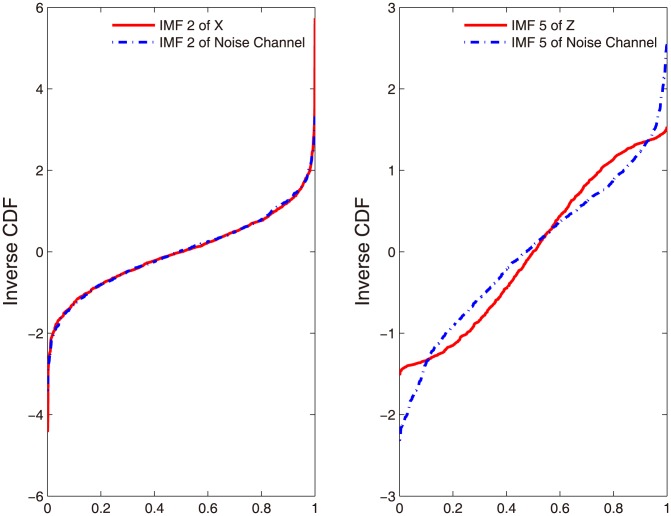
The inverse CDFs of IMFs, (A) IMF 2 of X and of the added noise channel; (B) IMF 5 of Z and of the added noise channel. Note that the IMF 5 of Z refers to the sine wave component of 26 Hz. It is clear that the significant information-bearing component (red solid in (B)) is clearly different from the IMF obtained from the noise reference (blue dash in (B)) in terms of inverse CDF, whereas the IMF without significant information content (red solid in (A)) almost completely overlaps with the noise IMF (blue dash in (A)).

Second, we construct a null distribution of the Wasserstein distance between the IMFs from the noise reference channels to establish the 95% confidence interval at each IMF scale. We also calculate the Wasserstein distance between IMFs obtained from the input data and those from the noise reference channels. To appreciate the difference of the Wasserstein distance obtained from the noise reference channels and the Wasserstein distance between the noise and the data, we show in Fig. 2 the comparisons of the inverse CDFs from two pairs of IMFs: one is an IMF without significant information (IMF 2 of X) compared to the corresponding IMF from the noise channel (IMF 2 of the noise); another is an IMF with significant information (IMF 5 of Z) compared to the corresponding IMF from the noise channel (IMF 5 of the noise). We note that, as the inverse CDF forms the basis for the estimation of Wasserstein distance (See Eq. 2 above), the examination of the inverse CDFs allows us to directly check if the Wasserstein distance is able to capture the subtle difference between the signal and the noise. As shown in Fig. 2, it is evident that the IMF without significant information (IMF 2 of X) matches well with the IMF obtained from the noise reference (Fig.2a), whereas the IMF with significant information (IMF 5 of Z, a component of 26 Hz) exhibits a clear difference from that of the noise reference (Fig. 2b). This example underlines the sensitivity of the Wasserstein distance in measuring the similarity between the reference IMF from the noise channel and the IMF from data.

Next, based on the 95% confidence interval obtained from the null distribution of the Wasserstein distance, we determine if an IMF obtained from the data is considered to be significantly informative (*p*<0.05). This is simply done by checking whether the Wasserstein distance to the noise reference is outside the 95% confidence interval. The results of the simulation are listed in Table 1. Clearly, all the information-bearing IMFs (in bold) are correctly identified, indicating that our approach is effective to identify the IMFs with significant information.

**Table 1:** Statistical identification of significant information-bearing IMFs by the Wasserstein distance.

IMF	distance between the signal and noise channels			distance between noise channels (95% confidence interval)
	X	Y	Z	
1	0.031	0.038	0.049	[0.024 0.054]
2	0.033	0.036	0.048	[0.024 0.059]
3	0.045	0.044	0.048	[0.024 0.069]
4	**0.239**	**0.256**	**0.238**	[0.032 0.108]
5	**0.230**	0.089	**0.232**	[0.035 0.142]
6	**0.228**	**0.228**	0.164	[0.047 0.201]
7	0.154	0.136	0.173	[0.065 0.321]
8	0.136	0.112	0.153	[0.049 0.243]
9	0.102	0.089	0.106	[0.017 0.328]
10	0.166	0.171	0.176	[0.023 0.281]
11	0.175	0,171	0.167	[0.016 0.405]
12	0.067	0,070	0.073	[0.005 0.225]
13	0.183	0.186	0.199	[0.010 0.524]

The results show that the designed IMF components (in bold) are all correctly identified.

**Figure 3:**
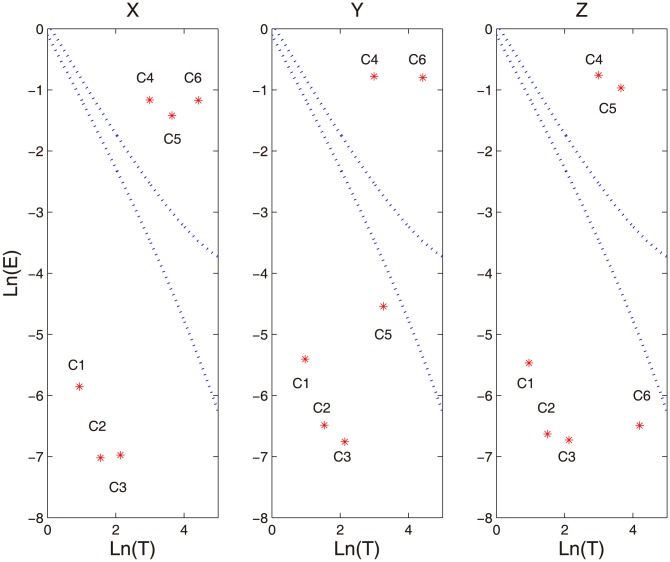
Statistical significance of first six IMFs for synthetic time series [X Y Z] by MEMD based on the method in [12]. In each panel, the energy of IMFs is plotted as a function of the logarithm of period, where the two dotted curves indicate 95% confidence intervals, corresponding to the upper and lower boundaries of the energy spread function, and each symbol ‘*’ refers to an IMF. The false identifications frequently occur in all three channels, e.g. all IMFs are identified significant in three channels.

As a comparison, we also apply the existing method [12], which is based on a presumed dyadic relationship between IMFs, to assess the significance of above obtained IMFs. Fig.3 shows the assessment of statistical significance of information content to the IMF components from channel X, Y and Z, respectively. In each panel, two dotted curves correspond to the upper and lower boundaries of the energy spread function [12], and each symbol ‘*’ denotes one IMF of the time series. In accordance with the method [12], if an IMF, denoted by ‘*’ here, lies outside two boundaries, it is considered as significant; otherwise noise. As shown in Fig. 3, many IMFs are not correctly identified. For instance, all IMFs of three channels are deemed as significant, while C1-C3 are known to retain no information.

#### Performance of the method in the presence of noise

**Figure 4:**
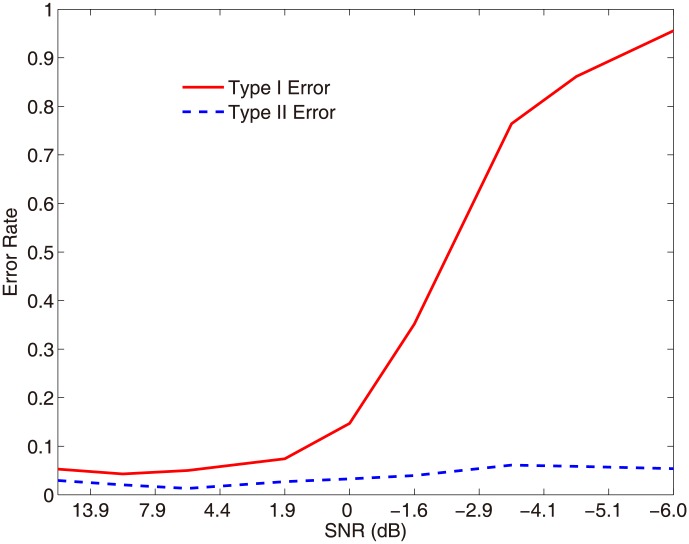
Performance of the proposed approach at different signal-noise-ratios (SNRs). SNR is systematically varied by changing the variance of the white noise superimposed in the trivariate data. At each SNR level, a data set of 100 trials is generated. The proposed method is applied to each trial to measure the signal identification performance, as quantified by both Type I error and Type II error.

Any neural data are inevitably contaminated by noise. The presence of noise can adversely impact the statistical analysis of data, thus impede our ability to extract meaningful information from noisy data. An important question is how our method performs on the data contaminated by noise. To address this question, we systematically vary the signal-to-noise ratio (SNR) in the above simulation via changing the variance of white noise superimposed in the trivariate data. At each SNR level, 100 trials of data are generated. We apply our method to each trial by following the same signal identification procedure above. The performance of the algorithm is measured as follows: failure to identify the true information-bearing IMF components (e.g., IMF 4 of Y) is referred to the Type I error, whereas falsely identification of the true information-free IMF components (e.g., IMF 5 of Y) is referred to the Type II error. Fig. 4 shows both Type I and Type II error rates over trials when our approach is applied to the data sets with the different SNRs. Our results show that both error rates are low at the high SNR region until it reaches 0, a point where the signal and the noise have equal power. As the SNR further decreases, Type I error increases rapidly, whereas Type II error remains very low. The results indicate that while the method fails to identify the information-bearing components with low SNR data, it is still able to tightly control the false identification of the information-free components. This is an appealing property, underlining the reliability of the method.

#### Mismatch of the noise contained in the data with the noise in the reference channels

In our approach, we have used the white noise as the reference channels. Clearly, the white noise is not the only type of noise encountered in practice. Ideally, the noise used in the reference channels should match the type of noise contaminated in the data – this is rare or almost impossible. Can the method still work when the noise contained in the data does not match the noise used in the reference channels? To answer this question, we use the same simulation set-up as described above, but with the pink noise (1/f noise) to replace the white noise superimposed in the trivairate time series. As such, the pink noise contaminated in the data does not match the white noise used in reference channels. Table 2 shows a typical identification result, where all information-bearing components are identified correctly. In addition, we repeat the analysis over 100 trials, yielding the excellent Type I error rate (<5%).

**Table 2:** Statistical identification of significant IMFs by the proposed method when the pink noise (1/f) in the data is different from the white noise in the reference channels.

IMF	distance between the signal and noise channels			distance between noise channels (95% confidence interval)
	X	Y	Z	
1	0.040	0.035	0.051	[0.025 0.058]
2	0.032	0.033	0.030	[0.024 0.052]
3	0.039	0.046	0.041	[0.024 0.098]
4	**0.231**	**0.242**	**0.230**	[0.033 0.099]
5	**0.235**	0.063	**0.237**	[0.031 0.133]
6	**0.208**	**0.208**	0.146	[0.044 0.172]
7	0.123	0.132	0.117	[0.058 0.224]
8	0.114	0.230	0.146	[0.040 0.262]
9	0.146	0.127	0.134	[0.027 0.403]
10	0.164	0.153	0.181	[0.031 0.552]

Our results show that the designed components (in bold) are all still correctly identified.

The observation that the method works well even when there is a mismatch of the noise contained in the data with the noise in the reference channels provides us strong confidence in application of the method for identification of information-bearing components in the data. In order to better understand why the method is robust to the noise mismatch, we perform the following simulation in which a trivariate time series is generated, consisting of three different types of noises with distinct characteristics: the white noise, the noise with positive long-range dependence (1/f noise) and the noise with negative long-range dependence [23].

**Figure 5:**
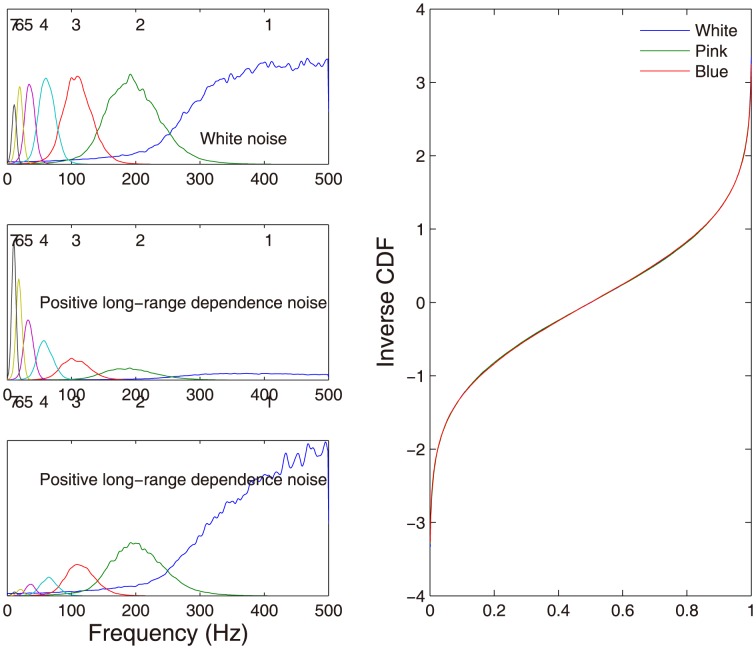
MEMD decomposition of the trivariate dada, consisting of three noise time series: the white noise (left top), the noise with positive long-range dependence (left middle) and the noise with negative long-range dependence (left bottom). The left panels show the power spectra of IMFs from the each noise (the sampling rate of 1000 Hz), in which the number denotes the order of IMF components. The right panel shows the inverse CDFs of IMF 3 from three different noise time series. Thanks to the rank-order statistics used in the estimation, there is an excellent match among three inverse CDFs, indicating that our approach is robust to the different types of noises.

The MEMD is then applied to the trivariate time series. Fig. 5 (left) shows the power spectra of IMFs 1-7 of three noises. We observe that their corresponding IMFs occupy very similar frequency responses similar to that of a dyadic filter bank, which is consistent with the previous study [11]. The existing method established by Wu & Huang [12] for assigning the statistical significance of IMF components is mainly based upon the characteristics of white noise (middle left) in that the product of the energy density of IMFs and the corresponding averaged period of IMFs is a constant. Clearly, it is not the case for other types of noises as each type of noise exhibits distinct energy distribution of its IMFs. Our procedure is based on the rank-order statistics to calculate the Wasserstein distance to measure the similarity between the IMF from data and the IMF from the noise reference, hence independent of the energy density of IMF of interest. We illustrate this idea in Fig. 5 (right) where the inverse CDFs of the IMF 3 obtained from three types of noises are compared. We can see from Fig. 5 that there is an excellent match among three inverse CDFs, indicating that our approach is robust to the different types of complex noises.

### Application to cortical field potential data

To demonstrate the usefulness of our approach to extract information-bearing IMF components from the neural data, we applied it to local field potentials (LFPs) data collected from visual cortex of macaque monkey while performing a visual illusion task, namely generalized flash suppressing (GFS).

**Figure 6:**
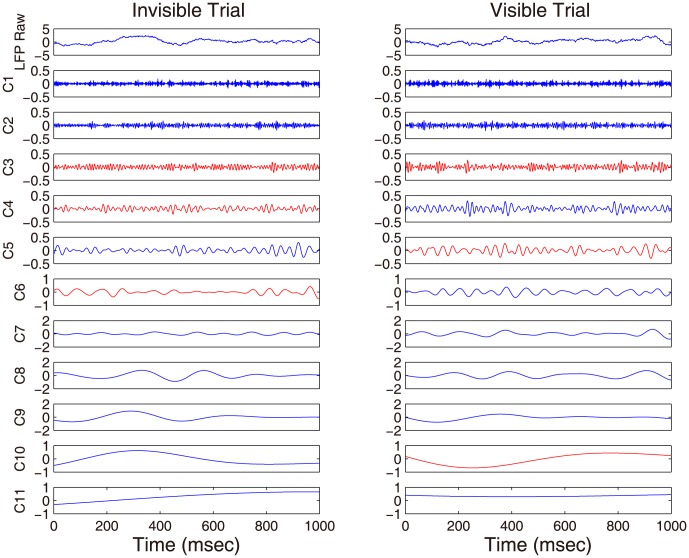
Examples of decomposition from two trials of LFP time series, one is from the invisible condition (Left), and another from the visible condition (Right). 0 indicates the surrounding onset. Our approach is able to identify the information-bearing IMFs, which are highlighted in red.

In this work, LFP after the onset of surrounding distractors from area V1 were used for illustration. Fig. 6 shows the results of decomposition for two single-trial LFPs, each from the invisible condition (shown in the left) and the visible condition (shown in the right), respectively. The top row of Fig. 6 corresponds to the raw LFP time series, which were adaptively decomposed into their inherent IMFs (shown as C1–C11 of Fig. 6). Importantly, the resulting IMFs are aligned both in number and in frequency scale in addition to superior frequency localization of IMFs. When our statistical identification procedure was applied on the IMFs, we found that C3, C4 and C6 in the invisible trial and C3, C5 and C10 in the visible trial (bold red curves in Fig. 6) were identified as significant. With our statistical identification procedure for all trials, we also observed that 1) the number of information-bearing IMFs is not exactly the same across trials, 2) not all the trials contain statistically significant IMFs, only 51 out of 61 trials have information-bearing IMFs identified, 3) the lower-order IMFs largely related to the deterministic trend and the first few IMFs related to the fast fluctuations (stochasticity) are often identified, and 4) the frequency localization of IMFs is improved, a finding consistent with the observation in [21]. These results make sense in that perceptual discrimination varies from trial to trail, so does neural activity. Therefore, we do not expect that every trial holds the same information.

**Figure 7:**
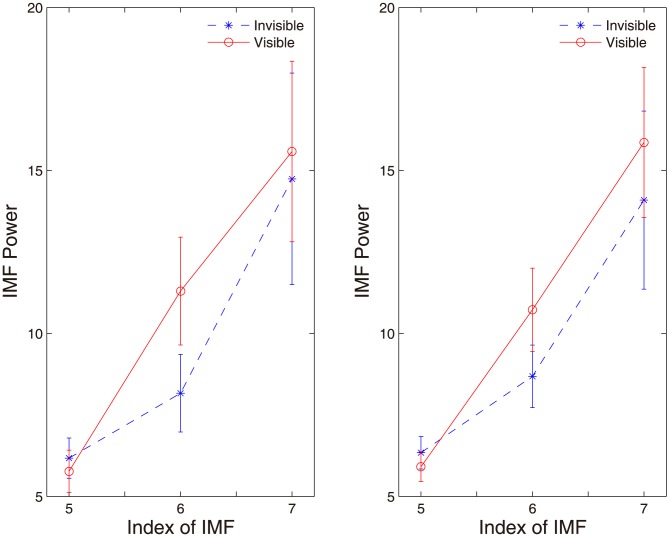
Discrimination of two perpetual conditions based on the identified information-bearing IMFs by (A) the proposed approach and (B) the method in [12]. It is clear that the IMF 6 in both methods show the most significant difference in power of two conditions, yet with a larger separation for the proposed method (*p*<0.01). The error bars denote the SEM.

Nonetheless, for neural data, unlike the simulations, we do not know the ground truth about the IMFs we have identified. To show the advantage of our approach over the existing method [12], we simply compare the difference in power of the identified information-bearing IMFs at three selected scales between two perceptual conditions (Invisible vs. Visible) over these two methods. The results are shown in Fig. 7. We found for both methods that 1) significantly larger power is observed in the visible condition than in the invisible condition, and 2) the 6^th^ IMF scale exhibits most significant difference in power between two perceptual conditions, yet with much less degree of discrimination for the method in [12].

**Figure 8:**
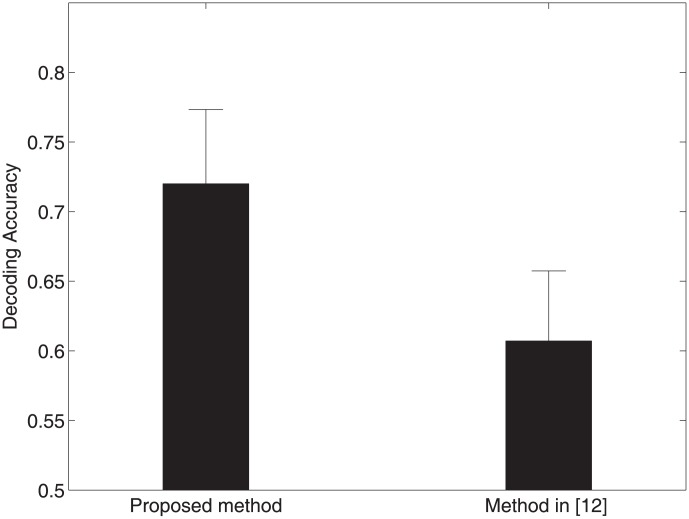
Comparison of decoding accuracy obtained by SVM with the proposed method (Left) and the method in [12] (Right). Clearly our approach provides higher decoding accuracy than that in [12].

To further demonstrate that the 6^th^ IMFs indeed contain significant information about the task, we take a decoding approach to predict a perceptual state from neural activity. In doing so, we compare the discriminative ability of the IMFs identified by our approach with the IMFs by the method in [12], in term of distinguishing two perceptual conditions. Support vector machine (SVM) is employed to decode two perceptual conditions based on the power of these IMFs. Decoding accuracy is calculated via leave-one-out cross-validation. Specifically, for a data set with *N* trials, we choose *N*-1 trials for training and use the remaining trial for testing. This is repeated for *N* times with each trial used for testing once. The decoding accuracy is obtained as the ratio of the number of correctly decoded trials over *N*. We found that our approach offers higher decoding accuracy of 72.0% than 60.7% obtained by the method in [12] (Fig. 8). The result indicates that the proposed approach is indeed able to extract the informative components of LFP data.

### Discussion and Conclusions

In this paper, we propose a novel method to focus on statistical identification of information-bearing IMF components within the framework of MEMD. In our approach, the white noises are added as extra channels to the original data for decomposition, which provide the reference for statistically identification of significant information-bearing IMFs. Wasserstein distance is employed to measure the similarity between the IMF from data and the IMF from noise reference channels. The rank-order statistics is used for efficiently estimation of Wasserstein distance, with confidence intervals assessed by the Monte-Carlo method. Computer simulations are performed to validate the proposed method. Application of our method is demonstrated on local field potentials data collected from visual cortex of macaque monkey while performing a generalized flash suppression task.

EMD and its improvements have been increasingly adopted in various fields of data analysis with great success, largely due to their adaptive data-driven nature. How to assign statistical significance of information content to IMF components from any noisy data is an unresolved question. Our method was driven by this important question. The key to our method is to distinguish how the IMFs obtained from the data differ from those obtained from the reference noise channels. This is assessed by the Wasserstein distance, a general distance function between probability distributions. Its confidence interval is established using a null distribution of the Wasserstein distance between the IMFs derived from reference noise.

To identify information-bearing IMF components, several approaches are currently available. First, some task-related domain knowledge as a priori can be used to determine if an IMF contains information. For example, the heartbeat (about 1 Hz) and respiratory signal (0.2–0.4 Hz) have different oscillatory frequency from the gastric slow wave (∼0.05 Hz) of interest in EGG recording; one can identify these components based on their distinct frequency characteristics [24]. Similarly in the study of visual attention, one can single out the gamma oscillations (30–80 Hz) from neural recordings to examine the attentional effect [25]. Second, the dyadic filter bank structure of white noise decomposition [12] can be exploited. Such a method is inadequate as the noise in the data is not necessarily white noise and the IMFs do not always adhere to the presumed dyadic relationship [26]. Third, delay vector variance (DVV) method uses the local predictability in phase space to address the issue of informative IMFs [27]. This method has been used to assess the degree of determinism and nonlinearity within the EEG data collected in steady state brain stimulations [28]. Our approach is in stark contrast to the existing methods as it uses noise to provide a reference to facilitate signal identification. The entirely data-driven nature of our method is attractive, thus making itself general applicable to broad data science.

When comparing two probability distributions, the Wasserstein distance is used to determine if two samples are drawn from different distributions. It is conceivable that other distance measures, ranging from the geometric distance such as L2-norm to information-theoretic measures such as Kullback–Leibler divergence, can also be used to assess the similarity between the reference IMF from noise channels and the IMF from data. Wasserstein distance is used as the similarity measure mainly due to its efficient estimation based on the rank-order statistics, which makes the method robust to outliers. In addition, confidence intervals for Wasserstein distance can be assessed via the Monte-Carlo method. Despite its promise, a number of limitations have to bear in mind while computing the Wasserstein distance. First, Wasserstein distance is a metric defined within the probability distribution space, which thus assumes the stationarity underlying the data. One solution to this problem is to estimate the Wasserstein distance over the time series in a short-time sliding window within which the data is considered being generated by an underlying (approximately) stationary stochastic process. The benefit for such a treatment is that it allows one to detect ephemeral signals contained in the data, which is not possible with the whole segment of time series. Second, in addition to identification of task relevant IMFs (e.g., IMF 6 in Fig.7), the similarity estimated by the Wasserstein distance often captures the higher-order IMFs (i.e., the first few IMFs) and the lower-order IMFs, as also observed by the DVV method [27]. Whereas the lower-order IMFs are largely deterministic and the trend-related, the higher-order IMFs contain information related to stochastic/nonlinear nature of the signal. Clearly, there is still room for improvement of Wasserstein distance to find the most informative IMFs. On the other hand, these IMFs must be interpreted with caution in real world applications.

Given the data-driven nature of EMD decomposition, the underlying probability distributions of IMFs from the noise reference are either unknown or extremely difficult to characterize. As such, the Monte-Carlo technique is employed to generate a null distribution for the Wasserstein distance in order to establish the confidence interval. This technique is computationally expensive. Other techniques such as the bootstrap resampling method [29] can also be used for estimating confidence interval when there is little knowledge of the underlying probability distributions. One advantage of this method is that, if more knowledge about the noise in the data or the underlying probability distribution is available, more advanced bootstrapping techniques may improve on the performance of the method.

Regarding the performance of the method, we have performed extensive simulations to examine 1) the noise contained in the data does not match the noise used in the reference channels (See Table 2); and 2) difference types of noise with distinct characteristics: the white noise, the noise with positive long-range dependence (1/f noise) and the noise with negative long-range dependence (See Fig.5 and the associated discussion). These simulations demonstrate that our approach is robust to both the noise mismatch and different types of noise. The robustness of our method is mainly due to the use of the rank-order statistics to estimate the Wasserstein distance, which is independent of the energy density of IMF of interest.

In closing, we have introduced a novel statistical procedure to determine the information-bearing components in neural data. Our method is entirely data-driven, robust to different types of noise used as the reference. We are enthusiastic that this new technique will prove itself of general value in the field of neural data analysis.

## Acknowledgments

We thank Dr Melanie Wilke for providing the data, which were collected at the laboratory of Dr Nikos Logothetis at Max Planck Institute for Biological Cybernetics in Germany.
